# Biocatalytic potential of *Brassica oleracea* L. var. botrytis leaves peroxidase for efficient degradation of textile dyes in aqueous medium

**DOI:** 10.1007/s00449-022-02820-x

**Published:** 2022-12-01

**Authors:** Umme Kalsoom, Haq Nawaz Bhatti, Kiran Aftab, Faiza Amin, Teofil Jesionowski, Muhammad Bilal

**Affiliations:** 1 Department of Chemistry, Government College Women University, Faisalabad, 38000 Pakistan; 2 Department of Chemistry, University of Agriculture, Faisalabad, 38000 Pakistan; 3 Department of Chemistry, Government College University, Faisalabad, 38000 Pakistan; 4 Institute of Chemical Technology and Engineering, Faculty of Chemical Technology, Poznan University of Technology, Berdychowo 4, 60965 Poznan, Poland

**Keywords:** Biocatalysis, *Brassica oleracea* peroxidase, Bioremediation, Textile dyes, Remazol turquoise blue, Drim Red CL4BN

## Abstract

Dye-contaminated wastewater discharge from textile and dye manufacturing industries is reported as a world worse water polluter due to the toxic and mutagenic behavior of dyes. Peroxidase, one of the key enzymes of oxidoreductases, is widely distributed in nature and has been currently exploited in industries for various applications. Widespread applications of peroxidases are associated with their nonspecific nature towards a wide spectrum of substrates such as phenols, aromatic amines, pesticides, antibiotics, and synthetic dyes. The present study explored the potential of ammonium sulfate precipitated partially purified *Brassica oleracea* L. var. botrytis leaves peroxidase for degradation of reactive textile dyes Remazol Turquoise Blue 133 G and Drim Red CL4BN. Various physico-chemical parameters such as pH (2–9), temperature (20–70 ℃), enzyme activity (3–24 U/mL), concentrations of H_2_O_2_ (0.4–1.4 Mm) and dye (10–100 mg/L) were optimized for enzymatic decolorization of both dyes’ solution. Studies revealed that maximum degradation (95%) of Remazol Turquoise Blue 133 G with peroxidase was achieved with 25 mg/L of initial dye concentration, in the presence of 0.8 mM hydrogen peroxide with 45 min of incubation time, at pH 3, 4, and 5, and 70 °C. Maximal decolorization (97%) of Drim Red CL4BN was obtained at pH 2.0, in 10 min of incubation time at 45 ℃ using *o*-dianisidine hydrochloride as a redox mediator. In conclusion, the findings illustrate the prospect of *Brassica oleracea* peroxidase to remediate dye pollutants and dye-based industrial effluents in a green technology theme.

**Graphical abstract:**

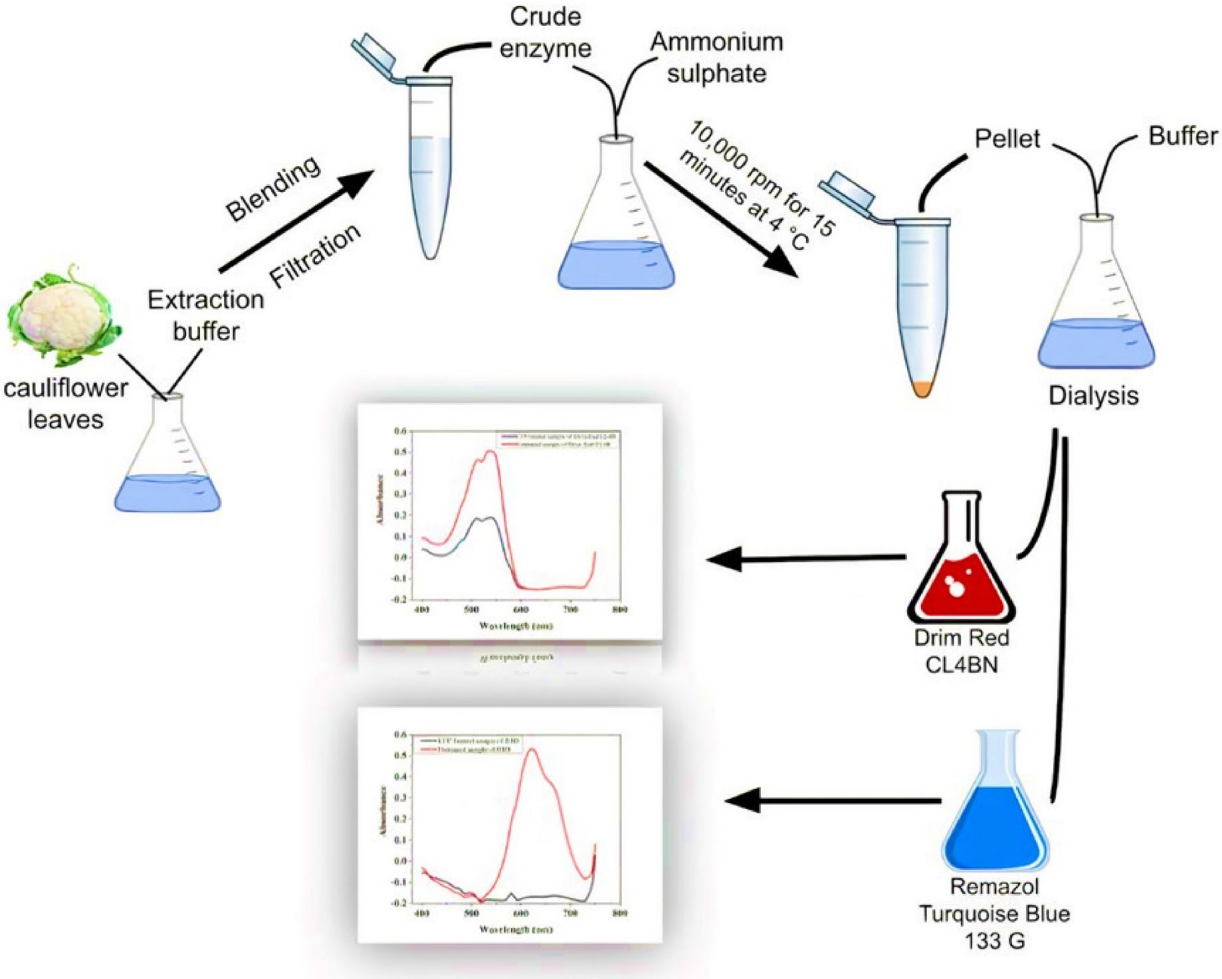

**Supplementary Information:**

The online version contains supplementary material available at 10.1007/s00449-022-02820-x.

## Introduction

Environmental protection against hazardous pollutants is one of the main concerns of biotechnologists and environmental researchers [27, 43]. Dye manufacturing and textile dyeing industries are considered worse water polluters as a consequence of key sources of imparting color pollution in water [37]. Dyes have low biodegradability [14] owing to their complex and stable molecular structures and exhibit persistent behavior towards light, temperature, and microbial actions. Colorants are highly visible in the water, even in small quantities, thereby reducing its transparency and decreasing the penetration of light, influencing the photosynthetic abilities of aquatic plants, the producers of the food web [44]. Moreover, synthetic dyes are known to have carcinogenic, mutagenic, and allergenic potential [24, 26]. Textile manufacturing and dyeing units discharge millions of tons of dye-contaminated wastewater into public drains, which are ultimately incorporated into rivers and seriously impact exposed organisms [13]. Therefore, it is crucial to eliminate dyes or convert them into less toxic metabolites before entering the aquatic ecosystem.

Numerous physical and chemical technologies, including photocatalytic, ozonation, coagulation/flocculation, adsorption, and inorganic catalysis, have been proposed for the remediation of wastewater of dye industries [17], 33]. All these technologies present some advantages and disadvantages. Some of these methods need expensive chemicals, and others produce concentrated sludge or toxic metabolites [16]. Therefore, an alternative, economically feasible, and environmentally friendlier technology is imperative for treating dye-contaminated wastewater [35]. Bio-based remediation technologies are gaining attention as promising alternatives all over the globe for decolorization of textile wastewater. Enzymatic bioremediation of industrial effluent is an emerging technology owing to its versatility and efficiency in the degradation of a wide variety of synthetic pollutants, including dyes [5].

**Fig. 1:**
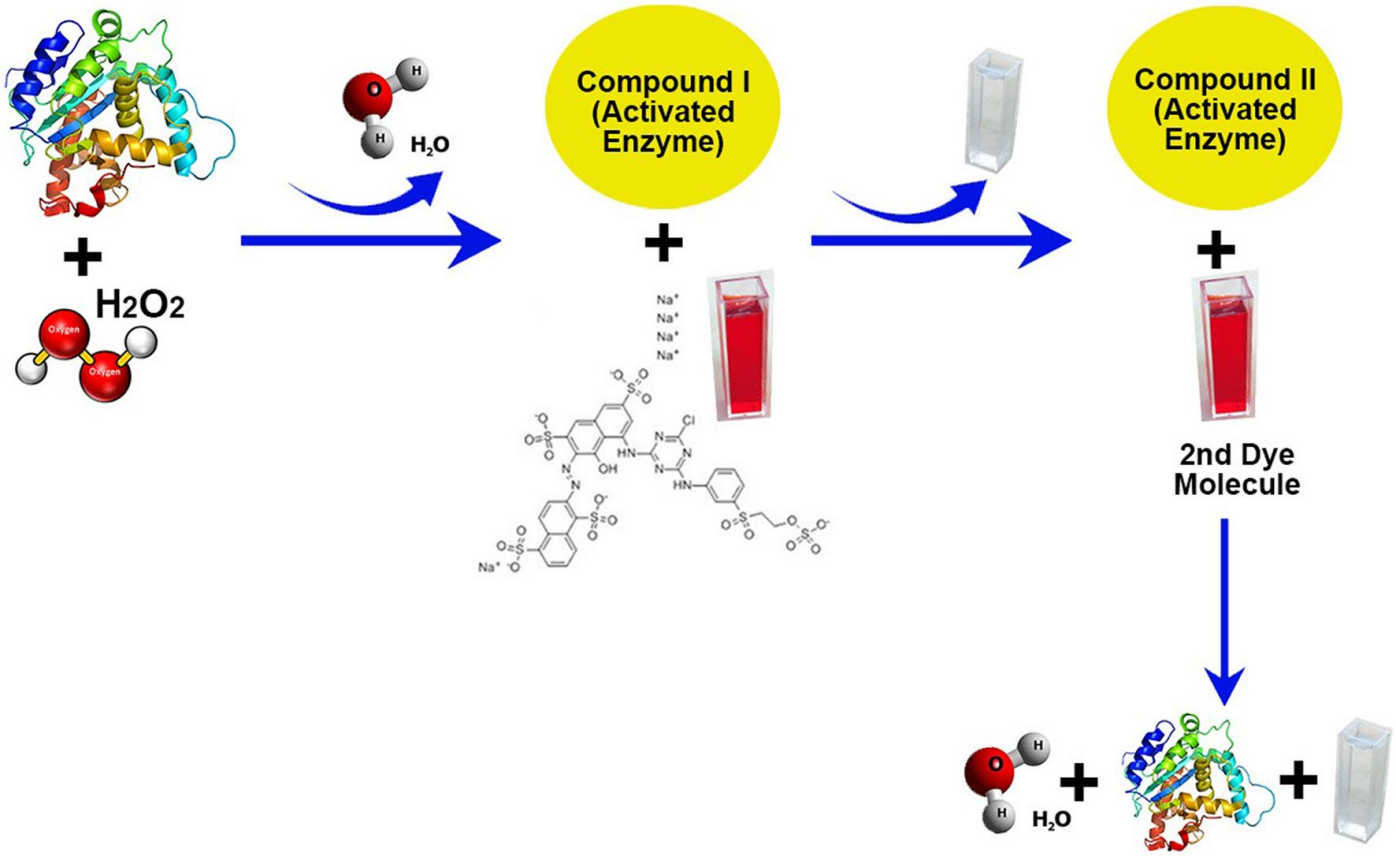
Graphical mechanism of catalytic cycle of peroxidase

Peroxidase (EC1.11.1.7; donor: hydrogen peroxide oxidoreductase), one of the key enzymes of oxidoreductases is widely distributed in nature and has been currently exploited in industries for various applications (Nataša et al. 2020). Widespread applications of peroxidases are associated with their nonspecific nature towards a wide spectrum of substrates such as phenols, aromatic amines, pesticides, antibiotics, and synthetic dyes [9, 10]. A wide array of biocatalytic interests in peroxidases are due to their catalytic potential to transform toxic substances into inoffensive or less harmful metabolites [8]. Plant peroxidases show a common mechanism of action for the degradation of aromatic compounds. In the first step, plant peroxidase coordinates with H_2_O_2_ and converts into catalytically active form compound I, which interacts with the dye molecule by accepting an electron and thus generates substrate-free radicle and compound II. This compound II is reduced by another substrate molecule into peroxidase native form and generates another free radical, water molecule. A graphical mechanism is explained in Fig. 1.

The use of peroxidase from vegetable leaves is a cost-effective and eco-friendlier way of dye removal from textile effluent. The peroxidase from cauliflower leaves is a better choice as it possesses much higher thermal stability and can be exploited for textile effluent treatment. Present study intends to demonstrate the catalytic potential of cauliflower leaves peroxidase for the biocatalytic detoxification of reactive dyes “Turquoise Blue 133 G and Drim Red CL4BN” in an aqueous solution.

## Materials and methods

### Chemicals

**Fig. 2:**
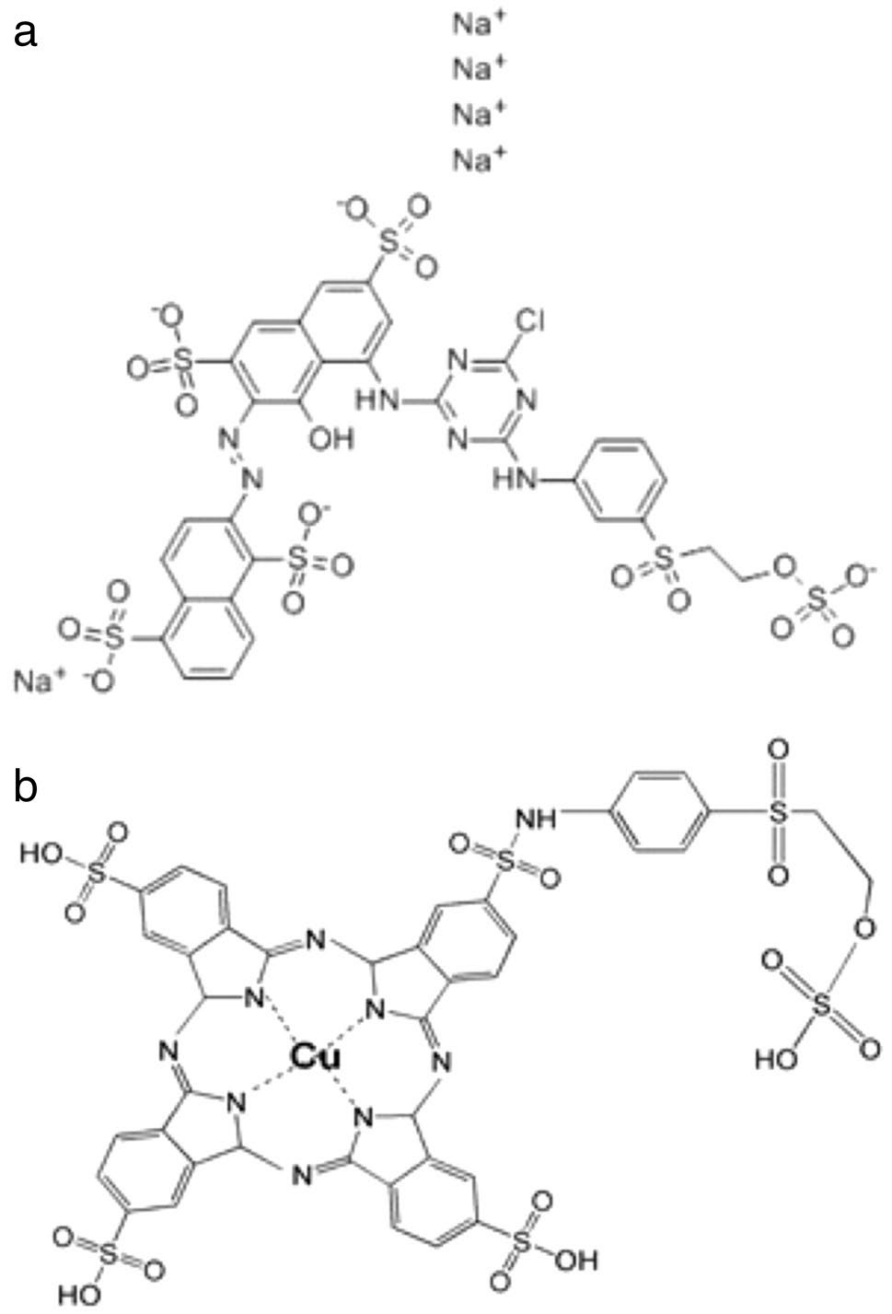
Molecular structure of **a** Drim Red CL4BN and **b** Remazol Turquoise Blue 133 G

The reactive dyes “Turquoise Blue 133 G and Drim Red CL4BN” (Fig. 2) were provided by True colors corporation. The chemicals acetone, hydrogen peroxide, calcium chloride were procured from Sigma Chemical Co. (St. Louis, MO, USA) and Merck (Germany) and were of analytical grade (> 98%). Catalyst peroxidase was extracted from Cauliflower (*Brassica oleracea* L. var. botrytis) leaves.

### Peroxidase extraction from cauliflower leaves

Cauliflower (*Brassica oleracea* L. var. botrytis) leaves were obtained from the fields of Faisalabad and thoroughly rinsed with distilled water to remove surface impurities. The enzyme was extracted by homogenizing the leaves in blending (for 15 min in short intermissions) in extraction buffer (0.1 M phosphate buffer) of pH 7.0 at a ratio of 1 g of leaves per 20 mL of extraction buffer. The crude extract of cauliflower peroxidase (CFP) was first filtered through four layers of cheesecloth and then using Whatman filter paper 1 under vacuum. Filtrate was assayed for peroxidase activity as well as for protein contents.

### Peroxidase activity

Peroxidase activity was monitored at 30 °C spectrophotometrically (Hitachi model U-2001) by following tetra guaiacol synthesis (*A*_max_ = 470 nm, molar absorptivity *ε* = 26.6 mM^−1^ cm^−1^) [28]. The composition of the reaction mixture is as follows: 1 ml of 0.1 M acetate buffer of pH 5.0, 1 ml of 15 mM guaiacol, 1 ml of 1.6 mM hydrogen peroxide, and 60 μl of enzyme extract. One unit of peroxidase activity (U) can be defined as the concentration of enzyme required to catalyze the conversion of 1 μ mole of guaiacol into the product in 1 min.

### Protein estimation

Total protein contents in the enzyme extract were estimated following the method of Bradford with bovine serum albumin (BSA) as a standard protein, as described below [11]. Stock solution of BSA (1 mg/mL) was prepared by dissolving 25 mg of BSA in 25 mL of deionized water. Standard concentrations ranging from 0.02 to 0.12 mg/mL were prepared by diluting deionized water. Bradford microassay was performed by incubating 0.1 mL BSA solution and 0.9 mL Bradford reagent for 5 min at room temperature and then determining the absorbance at 595 nm using a spectrophotometer (Hitachi model U-2001). The standard curve was then generated to get the standard factor (0.360) (Fig. S1).

### Ammonium sulfate purification of peroxidases

Powdered ammonium sulfate (168 g) was slowly dissolved to 300 mL of crude extract of cauliflower leaves to get 80% saturation. They were then left for 24 h at 4 °C and then subjected to refrigerated centrifugation at 10,000 rpm for 15 min at 4 °C. The pellets were redissolved in 30 mL of 0.1 M buffer, pH 7.0 and dialyzed against five changes of 0.1 M buffer (pH 7.0) to remove ammonium sulfate [9, 10]. The dialyzed solutions having peroxidase activity was used for decolorization of dyes.

### Selection of dyes for decolorization with CFP

Drim Red CL4BN and Remazol Turquoise Blue 133 G (RTB) dyes were selected for degradation with CFP. The molecular structures of Drim Red CL4BN and Remazol Turquoise Blue 133 G are shown in Fig. 1a, b, respectively.

### Decolorization of dyes with cauliflower peroxidase (CFP)

Reactive dyes Drim Red CL4BN and Remazol Turquoise Blue 133 G, Drim Black CL were treated with CFP. The initial dye concentration of 25 mg/L of both dyes were incubated individually with 12 U/mL CFP, 0.8 mM H_2_O_2_ for 1 h at 40 °C in a water bath shaker (Model # PA 9/250 U) at 60 rpm. The enzyme-treated dye samples were centrifuged before monitoring the color decrease at *λ*_max_ of each dye. The untreated dyes were considered as a control for the calculations of percent decolorization. The percent dye removal was determined by the following formula:

1 $${\text{Decolorization}}\left( \% \right) = \left[ {\left( {Ci - Cf} \right)/Ci} \right) \times 100]$$

where *Ci* is the concentration of untreated dye (control); *Cf* is the concentration of CFP-treated dye (after 1 h).

### CFP-catalyzed treatment of Remazol turquoise blue 133 G (RTB)

With the purpose of achieving maximum decolorization of RTB with CFP, various parameters of decolorization process were optimized.

#### Effect of CFP dose on decolorization process of RTB

To optimize the CFP dose needed for efficient dye degradation, 50 mg/L RTB in water was assayed with increasing concentrations of CFP (3–24) U/mL at 40 °C for 60 min using 0.8 mM H_2_O_2_ as co-substrate. Post-reaction solutions were centrifuged at 10,000 rpm for 5 min, and supernatant were used to measure the absorbance of RTB at wavelength *λ*_max_ 664 nm. The percent dye removal was estimated using above-mentioned equation.

#### Optimization of RTB concentration for decolorization process

Initial RTB concentration was optimized for maximum removal of dye by incubating different concentrations of RTB (12.5–100 mg/L) with 12 U/mL of CFP, 0.8 mM H_2_O_2_ for 60 min at 40 °C. Post-reaction solutions were centrifuged at 10,000 rpm for 5 min and supernatant was used to measure the change in absorbance of RTB at wavelength 664 nm. The percent dye removal was estimated using above-mentioned equation [1].

#### Optimization of incubation time for decolorization of RTB

The optimal incubation time was determined by incubating 25 mg/L of RTB with 12 U/mL of CFP and 0.8 mM H_2_O_2_ for an increasing time period (30–180 min) at 40 °C. The treated dye was centrifuged before measuring the absorbance (at 664 nm) of RTB. The percent decolorization was calculated, keeping untreated RTB as a control [1].

#### Optimization of pH for decolorization of RTB

The influence of pH on decolorization process was found by incubating the reaction mixtures containing 25 mg/L RTB, 12 U/mL of CFP, and 0.8 mM hydrogen peroxide for 1 h at 40 °C in the buffers of varying pH values (2–10) having 50 mM molarity. The controls (untreated RTB) were also prepared in buffers and used for determining percent decolorization [21].

#### Optimization of H_2_O_2_ for decolorization of RTB

In order to optimize the H_2_O_2_ concentration for maximum decolorization of RTB, dye solutions (25 mg/L prepared in 50 mM phosphate buffer of pH 5) were treated with a range of concentration (0.25–1.5 mM) of H_2_O_2_ and 12 U/mL of CFP for 1 h at 40 °C. Treated samples were centrifuged at 10,000 rpm for 5 min, and supernatant were used to measure the decrease in absorbance of RTB at wavelength 664 nm. The untreated dye prepared in buffer was used for calculations of percent decolorization.

#### Optimization of temperature for decolorization of RTB

The optimum temperatures of the dye decolorization reaction were determined by incubating 25 mg/L of RTB (prepared in phosphate buffer of pH 5) at various temperatures (40–75 °C) at the optimized conditions of enzyme dose (12 U/mL CFP) and H_2_O_2_ (0.8 mM). Samples were centrifuged and absorbance was noted at λ_max_ of RTB (664 nm). The untreated dye prepared in buffer was used to calculate percent decolorization [21].

### Decolorization of Drim Red CL4B with CFP

In order to get the maximum decolorization of Drim Red CL4BN with CFP, various parameters of decolorization process were optimized, as listed as follows:

#### Effect of *o*-dianisidine HCl on decolorization of Drim Red CL4BN

To investigate the effect of *o*-dianisidine HCl on degradation of Drim Red CL4BN, the parameters of decolorization process were optimized in the presence and absence of 3 μM *o*-dianisidine HCl.

#### Optimization of Drim Red CL4BN concentration for decolorization process

Standard solutions (12.5–100 mg/L) of Drim Red CL4BN were prepared in phosphate buffer (pH 5) and were incubated with an enzyme dose of 12 U/mL and 0.8 mM hydrogen peroxide for 1 h at 40 °C in the presence and absence of 3 μM *o*-dianisidine HCl. Post-reaction mixture was centrifuged at 10,000 rpm for 5 min, and supernatants were used to measure the decrease in absorbance of Drim Red CL4BN at wavelength 560 nm. The untreated dye solution was used for calculations of percent decolorization [21].

#### Influence of pH on CFP-catalyzed decolorization of Drim Red CL4BN

The optimized concentration (37.5 mg/L) of Drim Red CL4BN was prepared in 50 mM buffers of varying pH values (2–10). All the dye samples were incubated independently using 12 U/mL of CFP and 0.8 mM hydrogen peroxide in the presence and absence of (3 μM *o*-dianisidine HCl) for 60 min at 40 °C. Post-reaction solutions were centrifuged before monitoring the change in maximum wavelength (*λ*_max =_ 560 nm) of Drim Red CL4BN. The control solutions (untreated Drim Red CL4BN) were also prepared in buffers and used for the determination of percent dye removal.

#### Effect of CFP concentration for degradation of Drim Red CL4BN

To optimize the amount of CFP for maximum decolorization of dye, 37.5 mg/L of Drim Red CL4BN prepared in buffer (pH 3.0) was incubated with 0.8 mM H_2_O_2_ and with increasing concentrations of CFP (3–24) U/mL at 40 °C for 1 h with and without of 3 μM *o*-dianisidine HCl. All the samples were centrifuged, and absorbance were noted at λ_max_ of Drim Red CL4BN (560 nm). The percent decolorization was determined by calculating the difference in absorbance of the dye solution in a buffer of pH 3.0 without CFP enzyme (as a control) and absorbance of dye solution in a buffer of pH 3.0 as a control with CFP enzyme.

#### Optimization of incubation time for CFP-catalyzed degradation of Drim Red CL4BN

To optimize the incubation time for decolorization process, 37.5 mg/L of Drim Red CL4BN prepared in buffer (pH 3.0) was incubated with 12 U/mL of CFP and 0.8 mM H_2_O_2_ for an increasing time period (3–30 min) at 40 °C in the absence of 3 μM *o*-dianisidine HCl. All the samples were centrifuged, and absorbance were noted at *λ*_max_ 560 nm. The percent decolorization was determined by comparing the absorbance of dye solution in the buffer of pH 3.0 with and without CFP [19].

#### Optimization of temperature for CFP-catalyzed degradation of Drim Red CL4BN

The optimum temperatures of the dye decolorization reaction was determined by incubating 37.5 mg/L of Drim Red CL4BN (prepared in 50 mM buffer of pH 3) with 12 U/mL of CFP at different temperatures (35–75 °C) for 10 min using 0.8 mM H_2_O_2_. The post-reaction solutions were centrifuged prior to monitor at *λ*_max_ of Drim Red CL4BN (560 nm). The percent decolorization was estimated by calculating the difference in absorbance of dye solutions in a buffer of pH 3 with and without CFP (as a control) [19].

#### UV–visible spectral analysis of RTB and Drim Red CL4BN dyes

Dye degradation process was confirmed by UV–visible spectral analysis of untreated as well as peroxidase-treated samples of RTB and Drim Red CL4BN dyes.

## Results and discussion

### Remediation of reactive textile dyes with cauliflower peroxidase (CFP)

To degrade and detoxify polyaromatic hydrocarbons, biphenyls, and dyes, various reported enzymes (lignin peroxidase (LiP), manganese peroxidase (MnP)), phenol oxidases (laccases) and horseradish peroxidase (HRP) have attained attention for water treatment applications. In the present study, 64.31% and 21.1% decolorization was observed for Remazol Turquoise Blue 133 G (*λ*_max_ = 664 nm) and Drim Red CL4BN (*λ*_max_ = 560 nm) textile dyes, respectively, with cauliflower peroxidase (CFP) under the condition of [Dye] = 25 mg/L, [H2O2] = 0.8 mM, [CFP] = 40 U/mL. The literature revealed that soybean hulls peroxidase showed a degradation efficiency of 81.4% at 30 mg /L of methyl orange [15] and 41.0% of degradation of Remazol Turquoise G 133% was observed with commercial horseradish peroxidase [38, 39]. Calza et al. [12] observed the maximum removal of Orange I, Orange II, and Methyl Orange by soybean peroxidase within 2 h. Sekuljica and Mijin (2020) reported the 81.9% of Acid Violet 109 dye degradation using unpurified horseradish peroxidase extracted from soybean seeds extract. Amine functionalized superparamagnetic iron oxide immobilized horseradish peroxidase (HRP) showed 70% ± 4% maximum decolourization of Direct Blue 2 dye (Mandujano et al. 2018). The decolorization differences may be accompanied by the different structural features of individual dyes and the substrate binding specificity of peroxidase for different substrates. Therefore, the search for plant-based peroxidases bio-resource to degrade and remove recalcitrant dyes from textile effluents is striving for research to offer a continuous process for high-throughput operation.

### Decolorization of Remazol turquoise blue 133 G (RTB)

#### Optimization of CFP concentration for decolorization of RTB

**Fig. 3:**
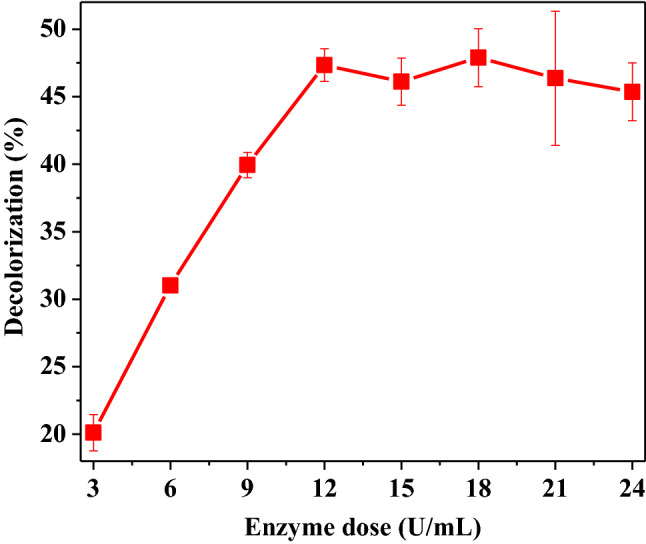
Optimization of CFP concentration for decolorization of RTB. Each value is expressed as the mean ± standard deviation (*n* = 3) of a triplicate analysis

Experiments were done to investigate the effect of concentration of CFP for degradation of RTB and the data are presented in Fig. 3. The results showed that the decolorization of RTB increased gradually with the increase in CFP concentrations and reached the maximum value at 12 U/mL of CFP concentration. No more increase in decolorization of RTB was observed with further increase in CFP concentration. The reason behind this is that no more dye molecules might be available to bind extra enzyme molecules. Thus, 12 U/mL of CFP concentration was assumed to be adequate for maximum decolorization of RTB dye (50 mg/L). The results obtained are consistent with earlier results [38, 39], in which maximum decolorization of 50 mg/L Remazol Turquoise G 133% was observed with a turnip peroxidase concentration of 10.83 U/mL. Balan et al. [3] optimized the 2.16 U/ mL laccase concentration to decolorize (96%) malachite green (95.80 mg/L) after 3.02 h.

#### Optimization of RTB concentration for decolorization process

**Fig. 4:**
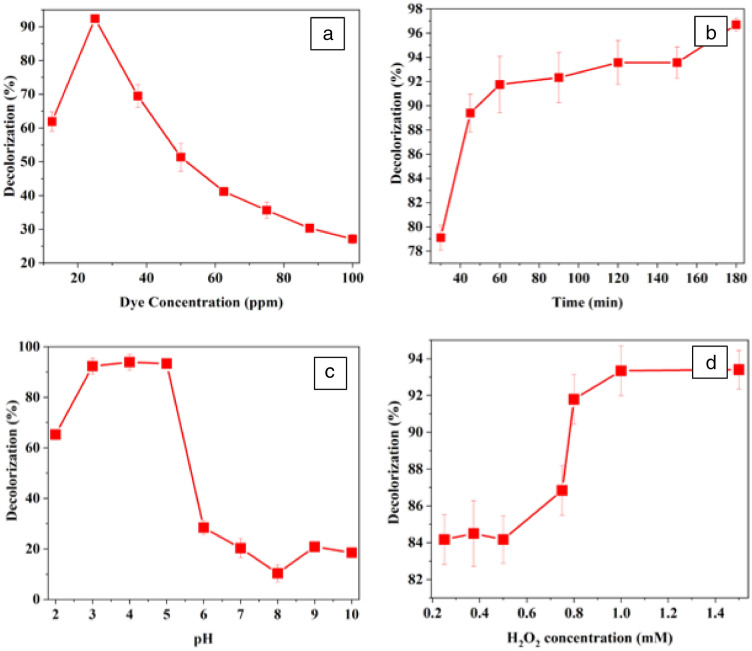
Optimization of **a** RTB concentration, **b** incubation time, **c** pH, and **d** H_2_O_2_ concentration (25 mg/L of RTB was treated with 12 U/mL of CFP in the presence of increasing concentrations (0.25–1.5 mM) of H_2_O_2_) for decolorization process. Each value is expressed as the mean ± standard deviation (*n* = 3) of a triplicate analysis

Amount of substrate has a substantial role in any enzyme-catalyzed reaction. Figure 4a demonstrates the role of amount of RTB on % decolorization. The dye decolorization was seemed to be effective up to 25 ppm of dye. Further increase in amount of dye caused low dye decolorization. The decrease in decolorization at high dye concentrations might be due to the presence of extra dye molecules in the reaction mixture because no more enzyme molecules are available to bind with extra dye molecules. The results are in agreement with previously published reports like maximum decolorization of fluorescein (almost 85%) with horseradish peroxidase was observed at dye concentration of 25 mg/L but a decrease in decolorization (35.7%) was seen with the increase in dye concentration upto 150 mg/L [34]. Similarly, Acid Black 10 BX decolorized maximally with horseradish peroxidase at 30 mg/L of dye concentration [32].

#### Optimization of incubation time for decolorization of RTB

Figure 4B shows the decolorization of RTB as a function of contact time with CFP. The experiments indicated that the dye decolorization increased as the reaction incubation time increases and reached to be optimum at incubation time of 45 min. After 45 min of reaction time, the increase in decolorization became negligible. The results are consistent with previous reported data. Optimum incubation time of Acid Black 10 BX (20 mg/L) with horseradish peroxidase for maximum degradation was found to be 45 min [32]. Remazol Turquois G 133% (50 mg/L) exhibited 50 min of incubation with turnip peroxidase for maximum decolorization [38, 39]. Similarly, Direct Blue-6 dye and its metabolites need 1 h of incubation with immobilized horseradish peroxidase to detoxify in the medium [4]. After 1 h of reaction time, the Direct Blue-6 dye and its degradation metabolites were removed enzymatically from the medium.

#### Optimization of pH for decolorization of RTB

Figure 4C explains the influence of pH of reaction medium on enzyme-catalyzed decolorization of RTB. It is obvious from the results that the decolorization of RTB is highly pH dependent: maximum decolorization was obtained at pH 3, 4 and 5 and decreased dramatically by increasing the pH. The results obtained are nearly similar to previously reported data. Cauliflower bud peroxidase catalyzed decolorization of some reactive and disperse dyes such as Reactive Black 5, Reactive Blue 4, Reactive Red 2, Disperse Black 9, and Disperse Orange 25 are pH dependent and showed significant decolorization in the pH range of 3–7; however, all these dyes showed maximum decolorization at pH 4 [19] 2. The impact of pH on decolorization of some reactive dyes (Reactive Orange 4, Reactive Orange 86, Reactive Blue 4, Reactive Blue 171, Reactive Blue 160, Reactive Yellow 84, Reactive Red 11, and Reactive Red 120) degraded with bitter gourd peroxidase was investigated. Most of the dyes showed maximally degraded within the pH range of 3.0–4.0. With the increase in pH of the reaction mixture up to 6.0, the rate of decolorization decreased for all the dyes. However, a small increase in the dye removal rate was noticed at neutral pH 7.0. Remazol blue degraded maximally with horseradish peroxidase at pH 2.5 (Bhunia et al. n.d.), while Disperse Red 343 showed maximum degradation with horseradish peroxidase at pH 5 [36]. Remazol Turquoise Blue G 133) showed maximum degradation with soybean peroxidase at pH 3.3 [29]. Maximum degradation of acid black 1 mediated by *Ziziphus mauritiana* leaves peroxidase was observed at an acidic pH of 4 and 5 [25]. Likewise, the optimal pH for soybean peroxidase catalyzed bioremediation of Methyl Orange and CI Direct Yellow 12 was observed to be 4 and 5, respectively [23].

#### Optimization of H_2_O_2_ for decolorization of RTB

H_2_O_2_ is a co-substrate of peroxidase, and dye degradation by peroxidase depends upon the amount of H_2_O_2_. Figure 4d shows the impact of H_2_O_2_ concentration on the decolorization of RTB. Studies showed that an increase in hydrogen peroxide concentration caused a gradual increase in dye decolorization and was found to be leveling off (93%) at 1 mM H_2_O_2_ concentration. However, 92% decolorization was achieved with 0.8 mM H_2_O_2_ concentration. These results agree with previously reported data. Jamal et al. [18] reported that the percent degradation of Disperse Black 9 and Disperse Red 19 with *Trichosanthes dioica* peroxidase first increased by increasing the amount of hydrogen peroxide and reached a maximum value of 0.8 mM. Further increase in H_2_O_2_ concentration (till 1.2 mM) did not cause any change in the dye decolorization [18]. Moreover, no hydrogen peroxide inhibition of the enzyme was observed with increased concentration of hydrogen peroxide. Similar findings are reported in the recently published literature on soybean, and potato peroxidase catalyzed degradation of Acid Violet 109 anthraquinone dye where biodegradation of dye was enhanced with an increase in hydrogen peroxide concentration from 0.1 to 1 mM, and no inhibitory effect was observed [42].

#### Optimization of temperature for decolorization of RTB

**Fig. 5:**
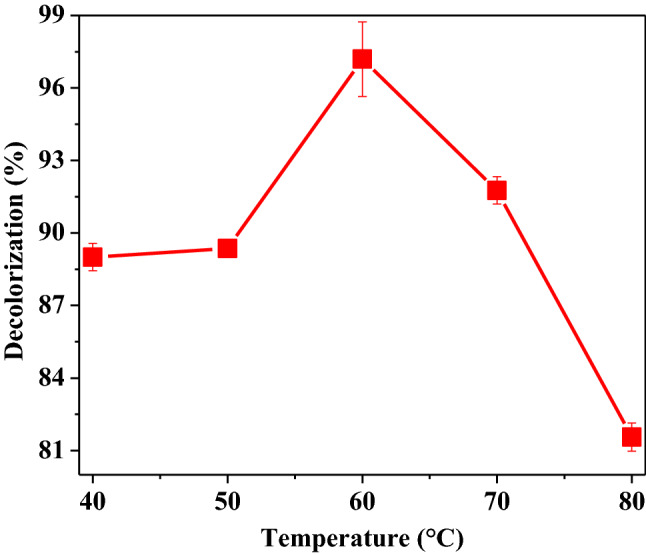
Optimization of temperature for decolorization of RTB. Each value is expressed as the mean ± standard deviation (*n* = 3) of a triplicate analysis

The impact of temperature on decolorization of RTB was also studied and the results are shown in Fig. 5. The enzyme was found to be highly thermostable and showed good decolorization efficiency at a wide range of temperatures from 40 to 80 °C with maximum decolorization observed at 70 °C. Maximum degradation of Disperse Red 343 with horseradish peroxidase was obtained at around 50 °C [36]. Soybean hull and potato peels peroxidase degraded anthraquinone dye C.I. Acid Violet 109 maximally at 38 °C; however, enzymes from these agriculture waste are active upto 70 °C and are able to apply for degradation of dyes at high temperatures [41].

### Decolorization of Drim red CL4B with CFP

#### Effect of *o*-dianisidine on decolorization of Drim red CL4BN

Redox mediators accelerate the catalytic potential and specificity of enzymes to a broad range of recalcitrant compounds. Several redox mediators have been reported to facilitate the activity of peroxidases; however, they are required in very high concentration in reaction mixture [30]. Karim and Husain [22] reported that a very small concentration (0.15 mM) of *o*-dianisidine might increase the peroxidase mediated oxidation of different aromatic amines from 2- to 65-fold. To explore the impact of *o*-dianisidine HCl on degradation of Drim Red CL4BN, the parameters of decolorization process were optimized in the presence and absence of 3 μM *o*-dianisidine HCl [22]

#### Optimization of Drim Red CL4BN concentration for decolorization process

**Fig. 6:**
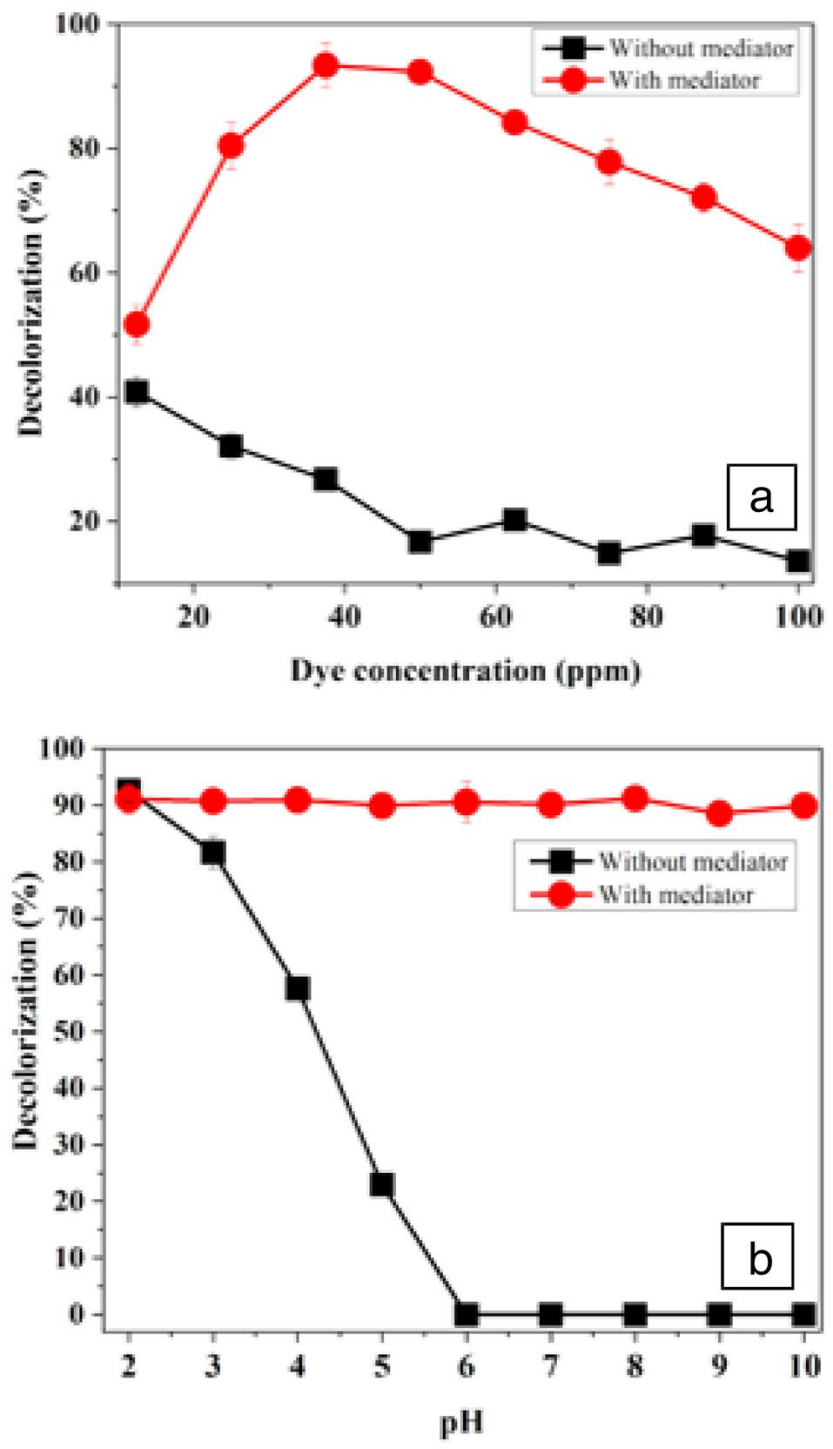
Optimization of **a** Drim Red CL4BN concentration, **b** pH ([H_2_O_2_] = 0.8 mM, [CFP] = 12 U/mL, [buffer] = 50 mM, [*o*-dianisidine HCl] = 3 μM, temperature = 40 °C) for CFP-catalyzed decolorization of Drim Red CL4BN. Each value is expressed as the mean ± standard deviation (*n* = 3) of a triplicate analysis

Experiments were done to optimize the concentration of Drim Red CL4BN in the presence and absence of 3 μM *o*-dianisidine HCl (Fig. 6a). The experiments showed that mediator had significant effect on decolorization of Drim Red CL4BN. In the presence of mediator, decolorization efficiency of the enzyme increased by increasing the concentration of dye up to 50 ppm and reached at maximum value (92%) and then reduced gradually with further increase in dye concentration, while in the absence of mediator maximum decolorization (40%) was observed at 12.5 ppm dye concentration and decreased with increase in dye concentration.

#### Optimization of pH for CFP-catalyzed decolorization of Drim Red CL4BN

The impact of pH on CFP-catalyzed decolorization of Drim Red CL4BN (in the presence and absence of *o*-dianisidine HCl) is shown in Fig. 6b. The results obtained revealed that the mediator increased the decolorization efficiency of the enzyme at a wide range of pH while in the absence of mediator decolorization decreased as the pH increases and reached to zero above pH 5. The data revealed that at low pH, the decolorization rate is nearly the same in the presence and absence of a mediator. Similar to the present study Boucherit and his coworkers (2013) also reported the highest decolorization rate (87%) for azo dye, Direct Yellow (50 mg/L) by *Cucurbita pepo* (courgette) peroxidase at pH 2.0, temperature 20 °C and H_2_O_2_ dose of 1 mmol/L.

#### Optimization of CFP dose for decolorization of Drim Red CL4BN

**Fig. 7:**
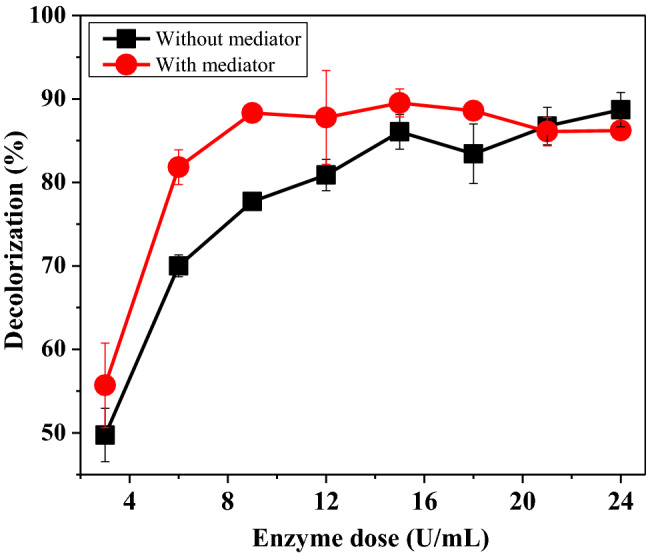
Effect of CFP dose for decolorization of Drim Red CL4BN. [Dye] = 37.5 mg/L, [H_2_O_2_] = 0.8 mM, [CFP] = (3–24) U/mL, [buffer] = 50 mM, pH 3, [*o*-dianisidine HCl] = 3 μM, temperature = 40 °C. Each value is expressed as the mean ± standard deviation (*n* = 3) of a triplicate analysis

The effect of CFP dose on the degradation of Drim Red CL4BN (with and without *o*-dianisidine HCl) was determined. At low pH, the effect of CFP concentration on decolorization reaction was nearly the same in the presence and absence of mediator. As can be seen from Fig. 7, 15 U/mL of enzyme concentration was found to be optimum in the absence, and 12 U/mL of enzyme concentration was observed to be optimum for maximum dye removal in the presence of a mediator.

#### Optimization of incubation time for decolorization of Drim Red CL4BN

**Fig. 8:**
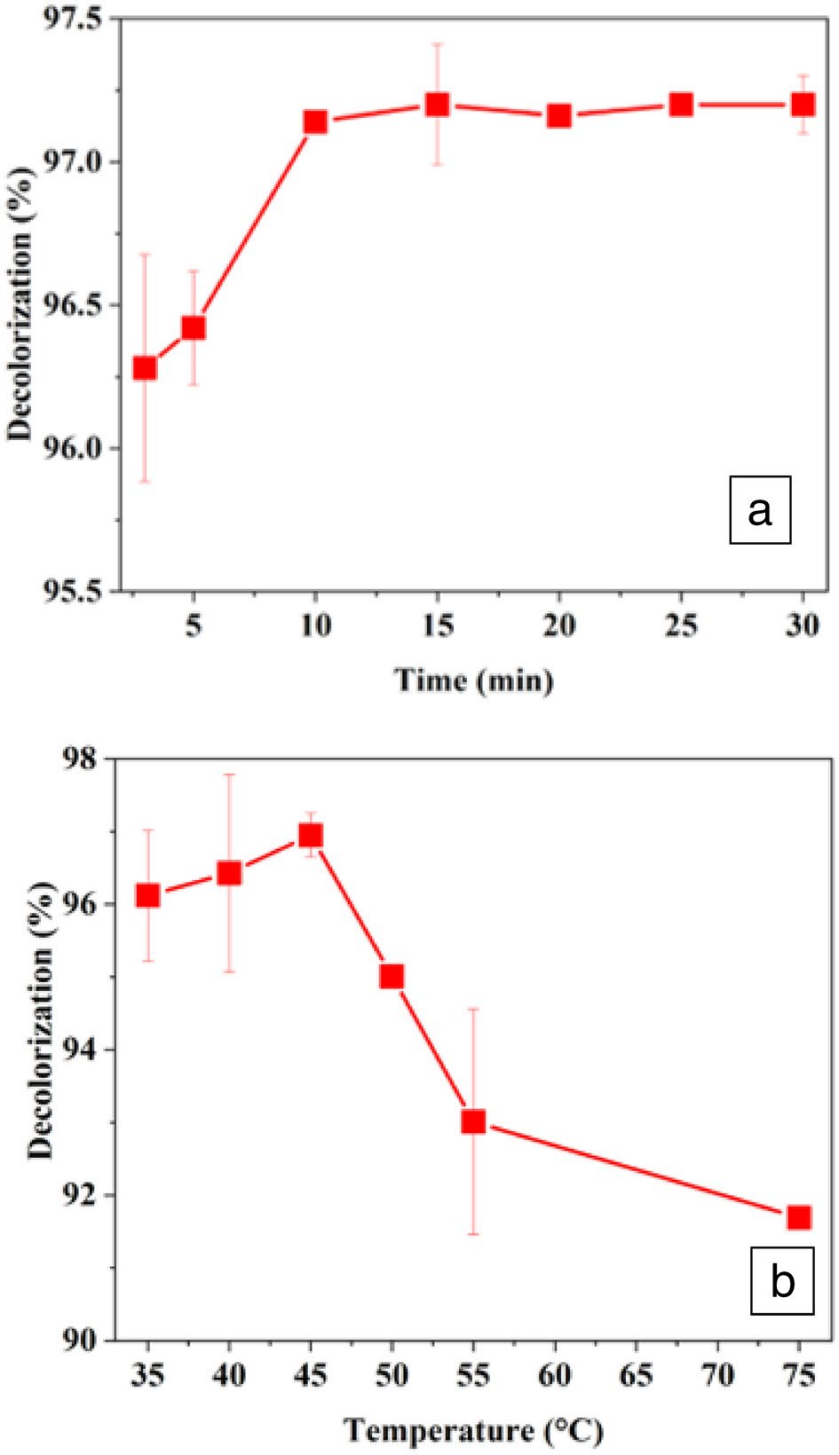
Effect of **a** incubation time and **b** temperature on decolorization of Drim Red CL 4BN. [Dye] = 37.5 mg/L, [H_2_O_2_] = 0.8 mM, [CFP] = 12 U/mL, [buffer] = 50 mM, pH = 3. Each value is expressed as the mean ± standard deviation (*n* = 3) of a triplicate analysis

At low pH values, the enzyme showed nearly the same decolorization efficiency with and without mediator, therefore, further experiments were performed in the absence of mediator at low pH. Figure 8a presents the effect of incubation time on decolorization of Drim Red CL 4BN in absence of *o*-dianisidine HCl. It was determined that 10 min of contact time is enough for 97% decolorization of Drim Red CL 4BN.

#### Impact of temperature on CFP-mediated decolorization of Drim Red CL4BN

The temperature activity profile for CFP-catalyzed decolorization of Drim Red CL 4BN is shown in Fig. 8b. Figure shows that maximum decolorization occurred at 45 °C; however, the enzyme was found to be very heat stable and showed good decolorization efficiency up to 70 °C. The findings are in agreement with previously reported data, e.g., Disperse Black 9 and Disperse Red 19 maximally decolorized at 42 °C with *Trichosanthes dioica* peroxidase [18]. Similarly eight reactive dyes (Reactive Orange 4, Reactive Orange 86, Reactive Blue 4, Reactive Blue 171, Reactive Blue 160, Reactive Yellow 84, Reactive Red 11, and Reactive Red 120) showed maximum decolorization at 42 °C with partially purified bitter gourd peroxidase [2]. Pirillo et al. also described the impact of three different temperatures (25, 45, 65 °C) on degradation of Flu Eriochrome Blue Black R and Fluorescein mediated by horseradish peroxidase. The data revealed that maximum decolorization of Fluorescein and Eriochrome Blue Black R was achieved at temperature 25 and 45 °C, respectively, while a small decrease in decolorization was observed with both dyes at 65 °C [34].

### UV–visible spectral analysis of decolorization of RTB and Drim Red CL4BN dyes

**Fig. 9:**
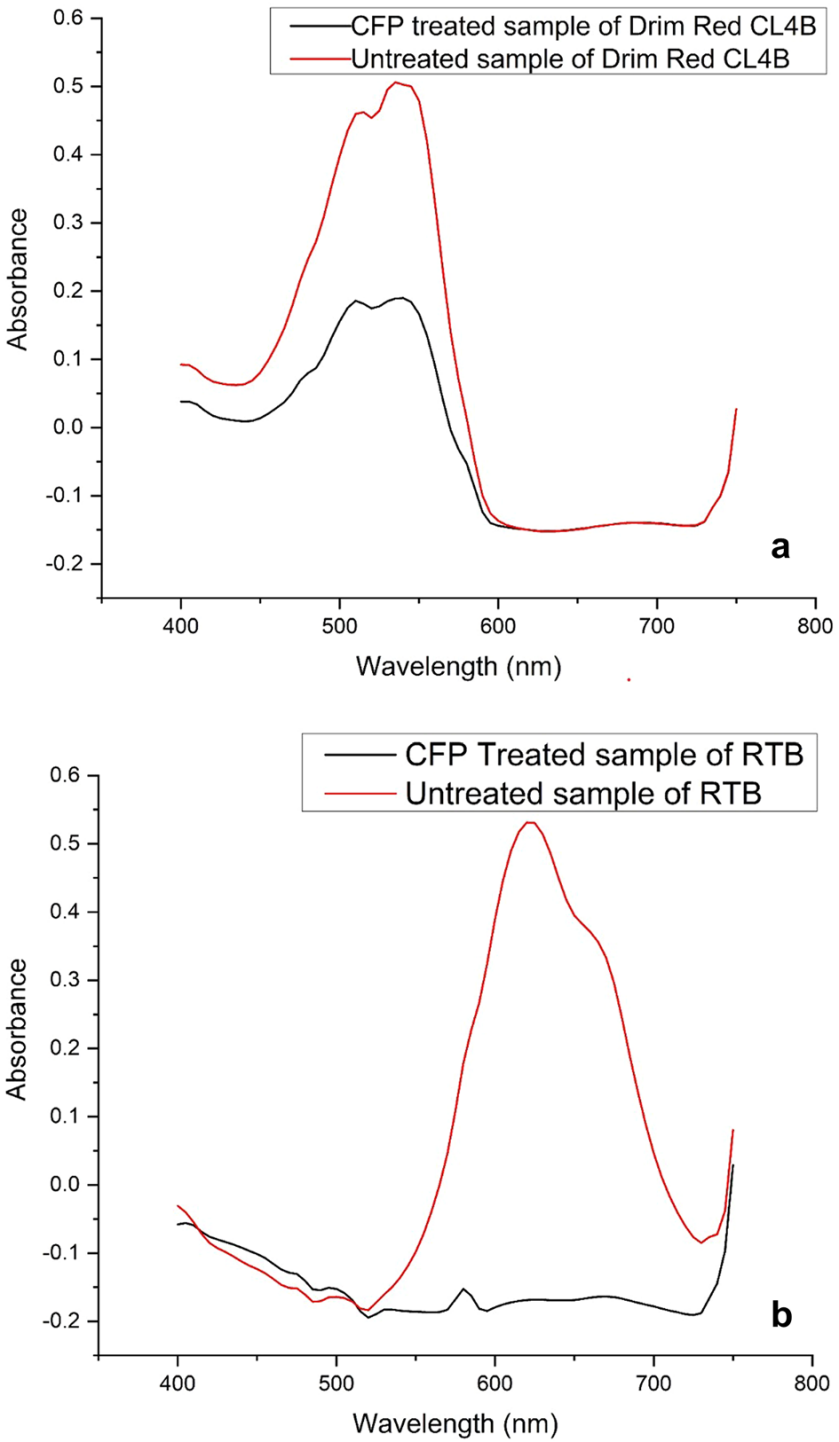
UV–visible spectral analysis of CFP treated and untreated **a** RTB dye, **b** Drim Red CL 4BN dye

Figure 9A and b shows the UV–visible spectral analysis of decolorization of RTB and Drim Red CL4BN dyes, respectively. A huge difference between absorption peaks of control and CFP-treated samples of RTB and Drim Red CL4BN indicated the removal of dyes in the treated samples. Figure 9a reveals the complete mineralization of RTB with CFP at optimized reaction conditions. In a recently published study, decolorization (94% and 100%) of a direct red dye with the free and immobilized Manganese peroxidase, respectively, was confirmed by UV/visible spectroscopy [20].

## Conclusion

This study reveals the prospect of *Brassica oleracea* L. var. botrytis leaves into a useful bioproduct, like peroxidase enzyme, and applied to biocatalytic degradation of reactive textile dyes Turquoise Blue 133 G and Drim Red CL4BN. The partially purified form of enzyme catalyzed the maximum decolorization of Remazol Turquoise Blue 133 G with an initial dye concentration of 25 mg/L in the presence of H_2_O_2_ after 45 min of incubation time at 70 °C. The finding that Turquoise Blue 133 G degradation efficiency by CFP remains unchanged over the broad range of pH 3–5 ensured its effectiveness for further study using real textile effluent. Moreover, the highest decolorization (96%) of Drim Red CL4BN was achieved at pH 2.0 in 10 min of incubation time at 45 °C in the presence of *o*-dianisidine HCl as a redox mediator. This study’s findings offer a prospective avenue for exploiting *Brassica oleracea* as a fascinating source of novel peroxidase enzyme with the capability of biodegrading textile reactive dye pollutants and dye-based industrial effluents in a green biotechnology agenda. Therefore, future research endeavor is a better understanding of the structure–activity relationship of dyes and enzymes-based bioreactors to commercialize the economical and eco-friendly biotechnology.

## Supplementary Information

Below is the link to the electronic supplementary material.

Supplementary file1 (DOCX 15 KB)

## Data Availability

Not applicable.
