# Supplementary material for: Biocatalytic potential of Brassica oleracea L. var. botrytis leaves peroxidase for efficient degradation of textile dyes in aqueous medium
Source: Bioprocess Biosyst Eng. 2022 Dec 1;46(3):453–65. doi: 10.1007/s00449-022-02820-x (PMC9950245; doi:10.1007/s00449-022-02820-x)
Supplement: Supplementary file 1 — Supplementary file1 (DOCX 15 KB) [file 449_2022_2820_MOESM1_ESM.docx]

**Fig. S1** Standard curve for protein estimation
